# Renal Transplant Outcome in Children with an Augmented Bladder

**DOI:** 10.3389/fped.2013.00042

**Published:** 2013-12-04

**Authors:** P. Lopez Pereira, Ruben Ortiz Rodriguez, Carlota Fernandez Camblor, María José Martínez Urrutia, Roberto Lobato Romera, Laura Espinosa, Enrique Jaureguizar Monereo

**Affiliations:** ^1^Paediatric Urology, University Hospital La Paz, Madrid, Spain; ^2^Paediatric Nephrologist, University Hospital La Paz, Madrid, Spain

**Keywords:** renal transplant, children, bladder augmentation

## Abstract

**Objective**: Studies evaluating renal transplant (RT) outcome in children who underwent an augmentation cystoplasty (AC) are contradictory and the current knowledge is based on studies with a limited number of patients. The aim of this study is to compare RT outcome between children who underwent AC and those without augmentation.

**Patients and methods**: A total of 20p who underwent an AC prior to the RT (12 with ureter and 8 with intestine) were enrolled in the study and were compared to a control group of 24p without AC, transplanted in the same time period (1991–2011). Data including; age at transplant, allograft source, urological complications, urinary tract infections (UTI) incidence, the presence of VUR, and patient and graft survival were compared between the groups.

**Results**: Mean age at RT and mean follow-up were 9.7 vs. 7.9 years and 6.9 vs. 7.9 years in the AC group and control group, respectively (NS). The graft originated in living donors for 60% of AC patients and 41.6% of the control RT patients. The rate of UTI were 0.01 UTI/patient/year and 0.004 UTI/patient/year in the augmented group and controls, respectively (*p* = 0.0001). In the AC group of 14p with UTIs, 10 (71%) had VUR and 5p out of 8 (62.5%) in the control group had VUR. In the AC group, of the 7p with ≥3 UTIs, 3 (43%) were non-compliant with CIC and the incidence of UTIs was not related with the type of AC or if the patient did CIC through a Mitrofanoff conduit or through the urethra. Graft function at the end of study was 92.9 ± 36.85 ml/min/m^2^ in the AC group and 88.17 ± 28.2 ml/min/m^2^ in the control group (NS). Graft survival at 10 years was also similar 88% in the AC group and 84.8% in controls. In the AC group 3p lost their grafts and 5 in the control group with respective mean follow-up of 10.6 ± 4.3 and 7.1 + 4.7 years.

**Conclusion**: There are no significant differences in the RT outcome between children transplanted with AC or without. However, recurrent UTIs are more frequent in the former group and these UTIs are related with non-compliance with CIC or the presence of VUR but, even so, UTIs will not lead to impaired graft function in most of the patients.

## Introduction

Lower urinary tract dysfunction (LUTD) can be responsible for renal failure in approximately 20% of ESRD children and just as this may lead to the destruction of their native kidneys, it can also adversely affect graft survival and function (1).

For a long time patients with LUTD were denied renal transplant (RT) because they were considered very high risk recipients but in the last few decades, the improvements in the medical management of LUTD as well as the development of novel surgical techniques (bladder augmentation, Mitrofanoff and Monti techniques, etc.) have improved these patients’ RT outcome.

Some patients with LUTD will need an augmentation cystoplasty (AC) to create a low-pressure and compliant reservoir that will protect the eventual renal allograft. However, some concerns have been raised that bladder augmentation in these patients may increase the risk of complications, predominantly urinary tract infections (UTI), urological complications, and allograft dysfunction and loss. Several studies have evaluated RT outcome in relation to AC in children (1–4) with contradictory results and our current knowledge is based on studies with a limited number of patients. This study compares RT outcome between children who underwent AC and those without augmentation to evaluate the impact of this technique.

## Patients and Methods

Of a total of 369 RT performed in 309 children (aged ≤18 years) in our department between 1985 and 2012, 20 patients had undergone AC before transplant. The causes of ESRD were posterior urethral valves (PUV) in 12p, neuropathic bladder in 7 and bladder agenesis in 1. The control group was made up of 24p with ESRD secondary to PUV. Both groups underwent transplantation during the same time period (1992–2011). In the AC group avoiding cystourethrography and urodynamic studies were performed in all patients when they were entering ESRD, except one (the patient with bladder agenesis). This study included a cystometrogram or flow-pressure study, electromyography, uroflowmetry, and measurement of post-voiding residual urine.

In the bladder augmentation group, this procedure was always performed prior to transplantation; AC was done with ureter in 12 patients, with sigmoid colon in 4, with ileon in 3 and with the ileocecal segment in 1. Twelve (60%) of the augmented patients had a Mitrofanoff conduit which was made with appendix in 7 and ureter in 5. Among the 8p without Mitrofanoff; 6 do CIC through the urethra and 2 do not need CIC to empty their bladders. In the augmented bladders, ureter reimplantation was performed into the native bladder in all cases following the Lich-Gregoir technique except for one, the patient with bladder agenesis.

In the control group (patients transplanted without an AC), 2p were on CIC through a continent catheterizable stoma made with appendix in one patient and with ureter in the other.

Urinary tract infections was defined by clinical symptoms including pyrexia, loin pain, or deterioration of graft function associated with a positive urine culture. Estimated glomerular filtration rate (GFR) was calculated using the Schwartz formula (5).

Data including age at transplant, allograft source, urological complications, UTI incidence, graft function and survival, and patient survival were compared between the augmented group and control group.

The immunosuppressive regime was similar in both groups because all of these patients were transplanted in the same period of time. It consisted of: antilymphocytic globulin or basiliximab induction + cyclosporine or tacrolimus + steroids + azathioprine or mycophenolate mofetyl.

This study was approval by the ethical committee of our hospital. Qualitative and quantitative variables were analyzed by chi-square and Student’s tests respectively. For analysis of the survival rate, the Kaplan–Meier method and Mantel–Haenszel log-rank test were used to compare the two groups. Statistical significance was considered at *p* < 0.05.

## Results

There were no significant differences between the augmented group and the control group in mean patient age at transplantation, mean follow-up and mean age at the end of the study. Grafts were obtained from cadaveric donors in 7 and from living related donors in 13 children in AC and from cadaveric donors in 14 and living related donors in 10 children, in the control group (Table 1). Two patients in each group had a previous failed kidney transplantation due to immunological causes.

**Table 1:** **Demographic characteristics of transplanted children with an AC and transplanted controls**.

Parameter	AC group	Control group	*p*-Value
Pre-transplant dialysis	55%	66.6%	NS
Mean patient (SD) age at RT	9.7 years (6)	7.9 years (5.3)	NS
LRD/CD	65/35%	42/58%	NS
Mean follow-up (SD)	6.9 years (5)	7.9 years (3.8)	NS
Mean patient age at end of study	16.7 years (5.5)	15.8 years (4.1)	NS

Surgical complications occurred only in two patients from the control group who presented a ureteral stenosis in the early postoperative period and were handled by doing a new ureteral reimplantation and placement a ureteral pig-tail. None of these complications affected graft function.

In the AC group, 7p (42%) had VUR grades III–IV: on one of the native kidneys in one of the patients, on the graft in two patients and on both kidneys in the remaining four patients. Three patients had a low grade VUR. Fourteen of the 20 patients in the AC group have had 37 UTIs (7p with ≥3 UTIs). Three (43%) of these patients with ≥3 UTIs during the follow-up were non-compliant with CIC.

We did not find any significant difference in the incidence of UTI between the type of bladder augmentation (ureterocystoplasty or enterocystoplasty) or whether the patient did CIC through the Mitrofanoff conduit or through the urethra. However, it is interesting that all the patients with VUR have had UTIs while only 4p (28%) with UTI did not have VUR.

In the control group five patients (21%) had VUR grades III–IV: on one of the native kidneys in 4p, and on both the graft and the native kidney in the last patient. In this group eight children have had 18 UTIs (3p with ≥3 UTIs). All patients with VUR have had UTIs and three without VUR have also had UTIs. None of the 16p who had never had UTIs had VUR on either their native kidney or on the graft.

The incidences of UTI were 0.01 UTI/patient/year and 0.004 UTI/patient/year in the augmented group and controls, respectively (*p* = 0.0001). Therefore, there is a significantly higher incidence of UTI in the augmented group than in the control group. However, UTI did not cause permanent graft function impairment in any patient and none of patients lost the kidney graft due to the UTIs.

Mean serum creatinine levels at the end of study were 1.1 ± 0.5 and 1.3 ± 0.5 mg/dl, in the AC group and the control groups, respectively (*p* = 0.4). The estimated GFR at the end of study was also similar for both groups; 92.9 ± 36.8 in the AC group and 88.1 ± 28.2 ml/min/1.73 m^2^ in control group (*p* = 0.6). Three renal grafts have been lost in the augmented group at a mean follow-up of 10.6 ± 4.3 years and five in the control group at a mean follow-up of 7.1 ± 4.7 years (*p* = 0.09). In all cases graft loss were due to chronic graft dysfunction.

**Figure 1:**
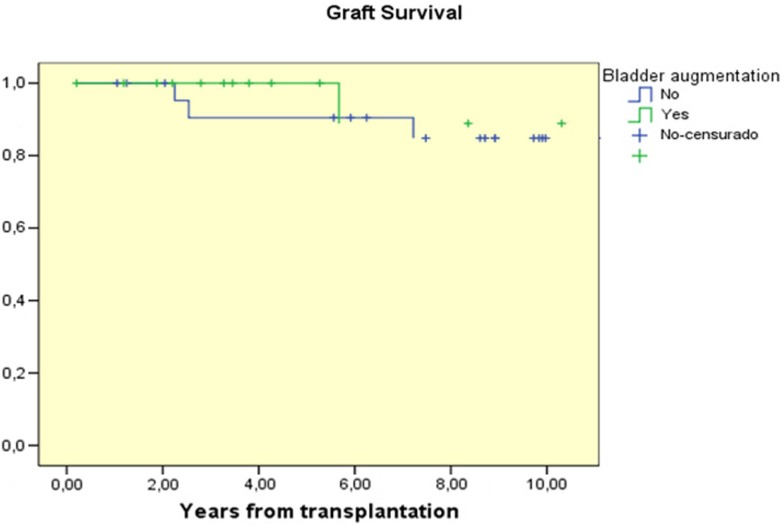
**Graft survival curves for children with AC prior to transplantation compared to those transplanted without AC**.

Patient survival in both groups was 100%. At 1, 5, and 10 years after transplant graft survival was 100, 100, and 88.9% in the AC group and 100, 90.5, and 84.8% respectively in the control group (NS) Figure 1.

## Discussion

Patients with LUTD are usually managed according to their degree of bladder dysfunction, as assessed by urodynamic studies and imaging and most of these patients are identified and treated very early, during infancy, to avoid their bladder dysfunction causing a deterioration of RF.

In those patients in whom bladder dysfunction is not diagnosed until renal failure, pre-transplant evaluation, and surgical management are necessary to plan the preparation of an adequate urinary reservoir with good capacity, compliance, and emptying before the kidney transplant. When a AC is required in these patients, ureterocystoplasty is preferable because it avoids the complications observed with gastrointestinal segments (metabolic acidosis, alkalosis, stones, intestinal obstruction, hematuria-dysuria syndrome, malignization, etc.). However, an adequately dilated ureter in a kidney without function is only feasible for a minority of patients. In this study 60% of patients had their bladder augmented with ureter.

Unfortunately, although the experience with bio-engineered implantable bladder substitutes that seek to provide low-pressure storage is promising it is not yet a reality (6).

When planning an AC in ESRD patients with LUTD, the patient’s ability to perform urethral catheterization must be evaluated before the surgery. If the patient is not able to do CIC through the urethra or this is painful, a Mitrofanoff or a Monti procedure would be necessary to allow adequate bladder emptying. In this study, 12p (60%) a continent catheterizable channel was necessary to do the CIC.

It has been suggested that patients with LUTD and an AC may present more urological complications and UTIs than other children receiving a transplant, affecting RT outcome. Several studies have evaluated RT outcome in relation to AC in children (1–4). Most studies (2, 7–9) did not find any differences in the urological complication rates after transplant between patients with LUTD, even when these patients had had their bladder augmented, and patients without LUTD. According to some authors, ureteral stenosis or fistulae could be more frequent when the graft’s ureter is not implanted in the native bladder (4, 10).

In our study only two patients in the control group presented a ureteral stenosis in the early postoperative period but neither of these complications had a repercussion on graft function and in all patients but one the ureter was implanted in the native bladder.

Recurrent UTIs are known to hasten deterioration of graft function and some authors have suggested that graft recipients with recurrent UTIs tend to have worse graft function although their graft survival does not differ from that of the general transplant population. Traxel et al. (2), in a recent study, compared 17 patients undergoing AC and subsequent RT with a control group of 17 on CIC who were transplanted without AC. They did not find significant differences in the incidence of UTI (0.07/year in the augmented group and 0.04 in the control group). Pereira et al. (3), compared a group of transplanted patients with augmentation (23p) to a group without augmentation (42p) and found an increased incidence of UTI in the augmented group. However, if only symptomatic UTI was considered, the same number of patients in each groups (4p) developed symptomatic UTI.

In our study there was a significantly higher incidence of UTI in the augmented group than in the control group (0.01 vs. 0.004 UTI/patient/year). However, UTI did not cause permanent graft function impairment in any patient and none of patients lost their kidney graft due to UTI. According to the results of this study, the risk of UTI in these patients is relates, in addition to immunosuppressive therapy, to the presence of native or graft kidney VUR and to non-compliance with CIC but it is not related to the type of AC or to if the patient did CIC through a Mitrofanoff conduit or the urethra.

Many studies have demonstrated that there are no significant differences in graft survival and function between patients with reconstructed bladders and those with normal bladders. Graft survival at 5 years ranges from 58 to 89% (2, 4, 10, 11), which is similar to the overall graft survival at 5 years from cadaveric donors (69.7%) and from living donors (82.6%), reported by NAPRTCS 2010 (12). Only the Basiri et al. study (4) found significant differences in graft survival between the augmented and the control groups (*p* = 0.03), but these differences were due to a higher incidence of acute and chronic rejection in the augmented group than in the control group (41 vs. 33%; 50 vs. 29%, respectively). In our study we did not find any significant differences in graft function and survival between the groups and the 10 years graft survival was similar in both groups, 88.9 and 84.8%, respectively (Figure 1).

Despite some limitations of the present study (it is retrospective, the number of patients is limited, only 2p were on CIC in the control group) we can conclude that RT can be successfully performed in children with LUTD and AC because there are no significant differences in urological complications, graft function, or survival between patients transplanted with AC or those without AC. However, recurrent UTIs are more frequent in the AC patients and these UTIs are related with non-compliance with CIC or the presence of VUR but, in most, the UTIs will not lead to impaired graft function.

## Conflict of Interest Statement

The authors declare that the research was conducted in the absence of any commercial or financial relationships that could be construed as a potential conflict of interest.
